# An Observational Study of OCD Patients Treated With Cognitive Behavioral Therapy During the COVID-19 Pandemic

**DOI:** 10.3389/fpsyt.2021.755744

**Published:** 2021-10-22

**Authors:** Vittoria Zaccari, Andrea Gragnani, Valerio Pellegrini, Tecla Caiazzo, Maria Chiara D'Arienzo, Antonella Magno, Giuseppe Femia, Francesco Mancini

**Affiliations:** ^1^Associazione Scuola di Psicoterapia Cognitiva (APC-SPC), Rome, Italy; ^2^Department of Human Sciences, Marconi University, Rome, Italy; ^3^Department of Social and Developmental Psychology Sapienza, University of Rome, Rome, Italy

**Keywords:** COVID-19, obsessive-compulsive disorder (OCD), obsessive-compulsive symptoms, cognitive behavioral therapy (CBT), Y-BOCS, adults

## Abstract

**Background and Objectives:** While the consequences of the COVID-19 pandemic for general mental health and the increase in anxiety and depression are clear, less is known about the potential effect of the pandemic on OCD. The purpose of this study is to collect new data to monitor the symptomatic status of patients with OCD during the period of emergency due to COVID-19 and to make a comparison between two psychodiagnostic evaluations.

**Methods:** Eleven OCD patients and their psychotherapists were recruited. All patients had a specific psychodiagnostic assessment for OCD (SCL-90; OCI-R; Y-BOCS self-report) performed between December 2019 and January 2020 (t0), and undertook cognitive behavioral therapy (CBT) and exposure and prevention of response protocol (ERP) before the lockdown. The psychodiagnostic assessment carried out at t0 was re-administered (t1) to all patients, together with a set of qualitative questions collected through an online survey. The respective therapists were asked to document the status of the therapy and the monitoring of symptoms through use of a semi-structured interview (Y-BOCS) and a qualitative interview. Non-parametric analyses were conducted.

**Results:** Patients reported a significant decrease in OCD symptoms. Data analysis showed a decrease in the scores across t0 and at t1 on the Y-BOCS (SR) total self-report, and on OCD symptoms' severity assessed by means of the OCI-r and SCL-90 r OC subscale, for 11 participants. Relating to the measures detected by psychotherapists, marginally significant improvements and lower scores were found in the Y-BOCS (I). An improvement in symptoms was noticed by 90.9% of the clinical sample; this was confirmed by 45.4% of the therapists, who claimed moderate progress in their patients.

**Conclusions:** The data collected through standardized measurements at two different times, albeit relative to a small sample, assume relevance from a clinical point of view. In the literature, some studies document the worsening of OCD. However, in many studies, the type of treatment, the detection time, and the intervention period are not well-specified. These results confirm the effectiveness of CBT/ERP as an elective treatment for OCD through a specific intervention procedure.

## Introduction

The COVID-19 pandemic had a significant impact on mental health in clinical and non-clinical populations (1–3). A wide range of studies has shown the impact on psychological distress in the adult clinical population (4, 5), in the general population (6–15), and in children and adolescents (16–20).

The pandemic has been associated with worsened mental health for those with pre-existing problems as well as new-onset mental health worries (21–23). The consequences for mental health and the increase of stress, depression, anxiety disorders, and suicide risk seem clear, as is well-documented in systematic reviews and metanalyses (10, 24–26).

The experience of long quarantine and isolation led to high levels of anger, stress, and confusion (27). Furthermore, during the pandemic, people were exposed to experience fear and fear linked to a greater likelihood of contamination, and were invited to implement protective behaviors. It is well-known that those who suffer from psychopathological anxiety engage in a series of safety-seeking, protective, and avoidance behaviors, or prudential reasoning, in order to avoid a threat, aspects that have a role in the maintenance of psychopathological symptoms (28, 29).

Although the consequences of the COVID-19 pandemic for general mental health and the increase in anxiety and depression are clear, less is known about the potential effect of the pandemic on obsessive-compulsive disorder [OCD; (30, 31)]. The increase in distress, concern, and fear has affected reactions to the present situation and exacerbated some existing psychiatric disorders (32, 33). In fact, some symptomatic domains have been triggered, typically anxiety or OCD (34, 35).

In this situation, the health impact of the COVID-19 pandemic on OCD cannot be overlooked. A growing body of research has shown how OCD is associated in some cases with a symptomatology that is highly sensitive to the fear and probability of contamination, with a perception of a greater possibility of becoming infected or infecting others, and with protective behaviors aimed at removing or neutralizing the possible risk of contamination (36–39), or preventing or neutralizing the fear of guilt related to irresponsibility (40–43, 48, 76) a specific mental state related to checking and cleaning compulsions (44). All these aspects were strongly conveyed in the period of emergency due to COVID-19.

The precautionary measures against COVID-19, such as hand washing, maintaining a high level of hygiene, and avoiding handshakes, may have triggered psychological distress in OCD patients, consequently increasing their symptoms. In this regard, it could be interesting to understand how the pandemic has triggered new or others fears, or if social isolation and restrictions have reinforced maintenance mechanisms such as avoidance behaviors, major doubt tendency, and major responsibility. Moreover, the constant condition of doubt, uncertainty, and fear of contagion may have caused an increase in prudential cognitive processes, mechanisms involved in the genesis and maintenance of the disorder (40, 41, 45–50). It is interesting to reflect on how this condition of uncertainty and alarm due to the pandemic has influenced OCD patients and so how the pandemic and the lockdown period may have affected OCD patients. However, two reviews have investigated the impact of the COVID-19 pandemic on OCD (30, 31) and the results are controversial.

A review by Sulaimani and Bagadood (30) analyzed all the empirical contributions on OCD, the most recent published in August 2020, and studies published from 2016 to 2020, and showed an increase in anxiety due to COVID-19, underlining that the associated prevention measures increase the severity of OCD symptoms. In their review, the authors highlight a worsening of OCD symptoms. However, it is impossible to generalize the results because the study refers only to the USA, China, India, and the UK. Another recent narrative review by Zaccari et al. (31) highlighted the presence of a small number of studies with controversial results. The authors underline that few studies have specified OCD subtypes and that samples cover a wide age range. Additionally, a large number of studies used different monitoring periods or quantitative measures, or did not use the Yale-Brown Obsessive Compulsive Scale (Y-BOCS), considered the gold standard for OCD evaluation (45a). Also, in most studies, the typology of treatment was not clear. Due to these differences and lack of information, it was difficult to compare and rely on the results.

Even if patients in some studies showed an increase in the severity and in the number of obsessions (51–54), OCD patients in others have been only minimally impacted by the pandemic (23, 55) or shown a slight decrease in symptoms (56). Moreover, regarding the typology of treatment, Zaccari et al. (31) showed that OCD patients were given cognitive behavioral therapy (CBT) treatment with exposure and response prevention (ERP) during the monitoring period in only two studies (54, 57).

Storch et al. (54) reported on clinicians' perceptions of the impact of the COVID-19 pandemic on individuals with OCD receiving ERP prior to and during the pandemic. From this study emerges a general worsening of OCD symptoms in patients (over one-third of patients) during the initial period of the COVID-19 pandemic despite continuing active treatment. These findings show that the COVID-19 pandemic represented a significant stressor for most patients with OCD, exerting a negative effect on symptomatology.

The results of the study by Kuckertz et al. (57) are different. Their typical program was an intensive program, including 2–4 h of ERP daily; four groups daily based on CBT and acceptance and commitment therapy (ACT); and meetings with a behavior therapist (2–3x/week), a family therapist (1x/week), and a psychiatrist (1x/week). Compared to the general population, their patients varied in the ways they were impacted by COVID-19, yet the majority experienced improvements in OCD symptoms despite the context.

Although CBT and ERP represent the elective treatment for OCD according to the National Institute for Health and Clinical Excellence guidelines (58), Zaccari et al. (31) reported that only two studies used this protocol. Over the last 30 years, different studies have confirmed the beneficial effect of CBT with ERP for OCD patients (59–62, 100).

Moreover, there is a lack of use of quantitative measures as the Y-BOCS, considered the gold standard for OCD evaluation. In fact, symptom change before and after treatment according to Y-BOCS assessments is the primary outcome measure in pharmacological and psychotherapeutic intervention trials for OCD [see meta-analysis by (63)]. As a result, national and international guidelines for evidence-based interventions for OCD largely depend on the reliability and validity of the Y-BOCS.

## The Current Study

This study aims to investigate the effects of the pandemic on OCD patients. In particular, the study aims to collect new data to monitor the symptomatic status of patients with OCD during the period of emergency due to COVID-19. At the same time, it aims to make a comparison between OCD subjects evaluated in the period December 2019–January 2020 and the current state. In accordance with the recent literature examined, we hypothesize that there is a general worsening of symptoms in OCD patients who presented a degree of impairment at the time of the initial evaluation through a difference in total scores in the different quantitative measures used for OCD symptoms. From the qualitative perspective, we expect that OCD patients and their respective psychotherapists will declare a worsening of their symptomatic status through a qualitative questionnaire. To achieve our purpose, we evaluated both patients' subjective perception of distress and the clinical judgment of psychotherapists through a self-report form and clinical interview.

## Materials and Methods

### Participants

For the purpose of this study, we recruited a sample of 11 adult Italian patients with OCD (male: 4; age: *M* = 34.55, *SD* = 7.88) who were undergoing treatment at the Center for Cognitive Psychotherapy in Rome but were not taking any medication, and their related psychotherapists. Diagnosis of OCD was made according to DSM-5 criteria (64) based on an extensive clinical examination and psychodiagnostic assessment.

Participants were recruited from the clinical assessment database belonged to the Center for Cognitive Psychotherapy in Rome. The selected patients were those undergoing CBT psychotherapy (with ERP) during the COVID-19 emergency period (March–December 2020). All patients had undergone previous treatment but had been evaluated at the symptom level in December 2019–January 2020.

Inclusion criteria for the study were as follows: being over 18 years of age; having a principal diagnosis of OCD, as diagnosed by an expert clinician using the Structured Clinical Interview for Diagnosis [SCID-5-CV; (65)]; having undergone a psychodiagnostic assessment with specific OCD quantitative measures and general symptomatology in the period December 2019–January 2020; having had CBT therapy with ERP during 2020; and reporting significant OCD symptoms at baseline [defined as a severe degree of impairment on the Y-BOCS ≥26 (66)]. Exclusion criteria included the following: significant physical or mental comorbid disorders (psychosis, schizotypy, borderline personality, mania, alcohol or drug abuse or dependence, impaired cognitive function); a current marked risk to self (self-harm or suicide); or dissociation symptoms (assessed on the basis of the first clinical evaluation with a clinical interview and psychodiagnostics assessment). Participant characteristics, gender, age, medications, and OCD subtypes for each subject are shown in Table 1.

**Table 1:** Clinical summary of participants.

**Participant**	**Gender**	**Age**	**OCD subtypes**
P 1	M	46	C and W
P 2	F	37	C and W, Ch
P 3	M	41	U
P 4	F	21	VO
P 5	F	40	C and W
P 6	F	42	AS
P 7	F	28	U
P 8	F	30	U, C and W, Ch
P 9	M	38	U, C and W
P 10	M	25	U
P 11	F	32	VO

*Gender: M, male; F, female; OCD, subtypes: Ch, checking; U, unacceptable taboo thoughts; C and W, contamination and washing; VO, Various Obsession; AS, all subtypes*.

### Procedure

Before recruitment to the study, we established the aims and the procedure, as well as the issues surrounding anonymity and privacy. Those who agreed to participate in the study were asked to complete a written informed consent and a battery of self-report questionnaires. No compensation was given for participation in the study. The procedure has been approved by the Ethical Committee of School of Cognitive Psychotherapy (Italy) and written informed consent from each participant were obtained before study initiation.

OCD patients who had undergone psychodiagnostic evaluations and clinical diagnosis between December 2019 and January 2020 (t0) were selected from the clinical center's database. The selected subjects had started a CBT intervention and treatment with ERP between January and February 2020. They were recruited between December 2020 and January 2021 again for a follow-up during the pandemic period (t1), undergoing the same psychodiagnostic assessment as at t0. Follow up data allow to monitor the symptomatic status of patients during the difficult period of COVID-19 pandemic.

Patients were assessed with respect to OC measures and symptomatology measures, and by qualitative questionnaires administered to their own psychotherapists and to the patients through an online survey prepared *ad hoc*. The assessment and scoring tests at t0 were rated by an expert psychodiagnostic clinician and at t1 by online survey, including a self-report measure and a qualitative report for patients and clinicians.

### Treatment

All subjects underwent a CBT intervention procedure according to international guidelines (67). The results obtained previously with this procedure were satisfactory: a clinically significant improvement was obtained in about 80% of all patients to whom this procedure was proposed. Furthermore, it is important to highlight that a very low rate of refusal of therapy and drop-out was found, at about 2% (67).

A crucial aspect of treatment is exposure with response prevention, consisting of a series of practical exercises for accepting consistently higher levels of threat. The underlying rationale is that if the patient increases the accepted level of risk, the effect is a lower investment in preventive activity and therefore less resistance to changing threat assumption (68–71). Alongside the exposure is a series of cognitive techniques and procedures aimed, in particular, at increasing motivation and collaboration in treatment and making the patient less vulnerable to the issues and mechanisms involved in maintenance of the disorder. The intervention procedure is divided into five phases.

The first phase starts after the preliminary assessment. It consists of reconstruction and sharing of the functioning scheme of the patient's disease. The purpose behind this phase is two-fold: to reconstruct the internal profile of the disorder (which allows rational planning of the therapeutic intervention), and to construct a good therapeutic alliance.

The second phase involves the possibility of modifying those beliefs that support the threat evaluation. The expected effect is the reduction of investment in threat prevention (72). Cognitive restructuring techniques—the probability pie technique and the cumulative probability technique—are used to reduce the probabilities of events (73), along with techniques to reduce responsibility (defense lawyer), and interventions for the normalization of forbidden thoughts (74), useful to reinforce an alternative to the beliefs that support valuations.

The purpose of the third phase is to promote factual acceptance, to help the patient enter the order of ideas that the threat, doubt and uncertainty can be accepted and should be accepted. This is achieved through modification of three different patient assumptions: the power to reduce risk, the advantage of investing in this direction, and the obligation to engage in risk reduction (75). This has a dual purpose. The first is to facilitate the acceptance of ERP exercises and increase the motivation to implement them. The second is justified by the fact that if a threat is accepted, the protective investment is reduced and, within this, the hyperprudent cognitive orientation, thus increasing the possibility that corrective information of the threat perception can really be taken into consideration by the patient (76, 77). At the same time ERP is introduced. The ERP is the most empirically effective CBT intervention (78). The procedure involves the combined application of two components: exposure and response prevention. It is appropriate to conceptualize the ERP as a series of practical exercises in accepting gradually increasing threat levels (79, 80).

The procedure used is the classic one in its different variants: gradual or prolonged exposure to the dreaded situations, with prevention of the responses frequently implemented by the patient (avoidance, and overt or covert rituals). The exposure predicts that the patient will be exposed to the feared stimuli, external or internal, for a time interval longer than he/she is normally willing to tolerate. Contact generally involves *in vivo* exposure with the anxiety-inducing stimulus according to a logic of progressive graduality; in some cases, due to precise therapeutic choices or depending on the specificity of the obsessive content, the exposure can also take place through an imaginative procedure. Prevention of the response, on the other hand, consists of interrupting the protective behaviors, also in this case for a longer time than usually elapses when the person postpones a ritual.

The fourth and fifth phases include action to reduce vulnerability to the OCD and are aimed to reduce the general disposition of OC patients to fear of guilt [(47, 81)]. This aim is pursued in two steps.

The first step (82) involves helping the patient to recognize the centrality that the fear of being guilty plays in their everyday life, focusing on situations in which this is activated and the quality of the internal dialogue, offering monitoring tasks to recognize the fear of being guilty, even at the body level. After this intervention the therapist helps the patient to decatastrophize the possibility of being guilty.

After that, the intervention is on the historical vulnerability working on elements of the patient's life history that have favored the onset of the disorder such as experiences, sensitizing episodes, factors predisposing to the specific disorder/problem. This phase's purpose is to make sensitizing experiences less relevant from an emotional point of view (83–86).

Finally, the last phase of treatment includes action to prevent relapses. At the end of the psychotherapeutic work, it is appropriate to discuss the possibility that the symptoms recur and define concrete interventions through which the patient can face them (75). Gragnani and Tenore (75) suggest sharing with the patient an alarm threshold beyond which it is convenient to contact the psychotherapist. Once the therapeutic objective has been achieved and an accurate phase of prevention of relapse finished it is possible to start the last part of the process: the conclusion of the therapy. It consists in retracing with the patient the phases of the work carried out and restructuring any dysfunctional beliefs on the end of the therapy.

### Measures

Participants were given a battery of online tests through the QuestionPro platform, guaranteeing privacy and assigning each of them an identification code associated with an alphanumeric code assigned by their psychotherapist. Demographic information was collected using an initial questionnaire created by the authors of the study.

Several self-report questionnaires were administrated to investigate the severity of symptomatology, as follows.

#### Obsessive-Compulsive Inventory-Revised

The Obsessive-Compulsive Inventory-Revised [OCI-r; (87, 88), Italian version by (89)] is a brief, 18-item self-report questionnaire designed to measure the presence and distress caused by obsessive compulsive symptoms on a five-point Likert-type scale ranging from 0 (not at all) to 4 (extremely). The instrument assesses symptoms on six different dimensions including washing, checking, ordering, obsessing, hoarding, and mental neutralizing (three items each). The OCI-r Italian version (89) showed good internal consistency as well as convergent, divergent, and criterion validity [alpha = 0.85]. In the present study, only the total score is used.

#### Yale-Brown Obsessive-Compulsive Scale

The Yale-Brown Obsessive-Compulsive Scale [Y-BOCS; (90, 91); Italian version by (92)] was used to assess severity of OCD symptoms. It is a widely used clinician- administered interview to assess the presence and the severity of OCD symptoms in adults. The Y-BOCS includes two sections: the symptom checklist (Y-BOCS-SC) and the severity scale (Y-BOCS-SS). The symptom checklist is a clinician-rated checklist designed to guide a structured interview to determine the target symptoms for treatment. The severity scale consists of 10 items that assess the severity of obsessions (five items) and compulsions (five items) using a five-point Likert-type scale (from 0 to 4; the total score of the scale may range between 0 and 40). The final rating is based on the clinical judgment of the interviewer. In the present study, the Y-BOCS interview was administered by psychotherapists in parallel with online administration of the self-report form [Y-BOCS-SR; (93)]. Only the total score of Y-BOCS and the obsessions and compulsions subscales both in the self-report form to the patient and in the version by the clinicians (their psychotherapist) are used. The scales showed strong internal consistency for the total score and each subscale [α = 0.89–0.93; (94)].

#### The Symptom Checklist 90-Revised

The Symptom Checklist 90-Revised [SCL-90-R; (95); Italian version by (96)] is self-report questionnaire designed to measure general psychological symptoms and psychological distress. It contains nine subscales—somatization (SOM), obsessive-compulsive (O-C), interpersonal sensitivity (I-S), depression (DEP), anxiety (ANX), hostility (HOS), phobic anxiety (PHOB), paranoid ideation (PAR), and psychoticism (PSY)—and the nine frequently used subscales provide symptom profiles. It is a five-point Likert scale ranging from 1 to 5, where 1 = “not at all,” 2 = “a little bit,” 3 = “moderately,” 4 = “quite a bit,” and 5 = “extremely.” Thus, a higher score reflects a higher frequency or intensity of symptoms. The Global Severity Index (GSI) is equal to the average of all nine subscale scores. The internal consistency coefficients for the nine subscales were 0.76–0.90. In the present study, only the OC subscale is used.

### Qualitative Questionnaire

In order to test our hypotheses, two questionnaires (one for psychotherapists and the other for patients) were created specifically for the purposes of the study and investigated a set of variables: (a) general information; (b) the frequency of psychotherapy during the pandemic and lockdown period; (c) possible suspension of psychotherapy; (d) evaluation of a worsening of symptoms; (e) evaluation of an improvement in symptoms; (f) appearance of new obsessions or compulsions; and (g) perception of how much the psychotherapy in progress has represented a protective factor against worsening or relapse.

### Statistical Analyses

Given the small number of participants, data were analyzed using a non-parametric test. Specifically, we implemented the Wilcoxon Signed Ranks Test for non-parametric comparison of the scores between the baseline (t0) and during treatment (t1) to show y participants on the following measures: total score of Y-BOCS-SS, obsessions and compulsions subscales, total score of OCI-r, and SCL-90 OC subscale. Analyses were conducted using the Statistical Package for Social Science (SPSS 25, Inc., Chicago, IL).

## Results

### Descriptive Statistics

In a preliminary exploration of the data, we computed the descriptive statistics of the investigated variables at each measurement time (t0 and t1). Table 2 shows the resulting means and standard deviations. Because of the reduced size of the sample under examination, descriptive analyses were enriched by computing the median of participants' scores on each measure and the associated interquartile range. This latter was considered an indicator of data dispersion from the central tendency.

**Table 2:** Descriptive statistics.

**Measures**	**Statistics**	**t0**	**t1**
Y-BOCS (SR) total	Mean	30	16
	Ds	5.9	8.7
	Median	30	17
	IQR	9	14
Y-BOCS (SR) Obs.	Mean	15.2	8.5
	Ds	2.8	4
	Median	15	9
	IQR	4	5
Y-BOCS (SR) Comp.	Mean	14.8	7.5
	Ds	3.4	4.9
	Median	15	8
	IQR	6	8
Y-BOCS (I) total	Mean	24.8	20.1
	Ds	6.9	8.2
	Median	21	21
	IQR	13	8
Y-BOCS (I) Obs.	Mean	13.4	10.5
	Ds	3.4	3.5
	Median	12	11
	IQR	5	5
Y-BOCS (I) Comp.	Mean	11.3	9.4
	Ds	4.5	5.2
	Median	11	10
	IQR	8	7
OCI-r	Mean	21.5	16.4
	Ds	15.5	14.3
	Median	16	8
	IQR	14	23
SCL-90 r OC	Mean	1.8	1.2
	Ds	0.9	1
	Median	1.6	0.9
	IQR	1.2	1.6

*Means; Ds, standard deviations; IQR, median and Inter-Quartile Range; Y-BOCS, Yale–Brown Obsessive–Compulsive Scale, and the obsessive (Obs.) and compulsive (Comp.) sub-dimensions; OCI-r, Obsessive–Compulsive Inventory Revised; SCL-90 r OC, Symptom Checklist 90-Revised Obsessive-Compulsive subscale; SR, self-reporting; I, interview. Table reports comparisons between the baseline (t0) and follow-up (t1)*.

### Wilcoxon Signed Ranks Test

As previously mentioned, the small number of participants allows investigation of our hypotheses by means of non-parametric analyses. Thus, we implemented a Wilcoxon Signed Ranks Test for non-parametric comparison of the investigated OCD symptomatology between the t0 and t1 assessments (see Table 3).

**Table 3:** Wilcoxon Signed Ranks Test for study variables.

	**Comparisons**	**Negative ranks**	**Positive ranks**	**Ties**	**Total**	***Z***	***p***
Y-BOCS (SR) total	t0 vs. t1	11	0	0	11	−2,937	0.003
Y-BOCS (SR) Obs.	t0 vs. t1	11	0	0	11	−2,941	0.003
Y-BOCS (SR) Comp.	t0 vs. t1	11	0	0	11	−2,937	0.003
Y-BOCS (I) total	t0 vs. t1	9	2	0	11	−1,914	0.056
Y-BOCS (I) Obs.	t0 vs. t1	8	2	1	11	−2,002	0.045
Y-BOCS (I) Comp.	t0 vs. t1	7	4	9	11	−1,254	0.210
OCI-r	t0 vs. t1	9	2	0	11	−2,229	0.026
SCL-90-r OC	t0 vs. t1	8	2	1	11	−2,250	0.024

*Y-BOCS, Yale–Brown Obsessive–Compulsive Scale, and the obsessive (Obs.) and compulsive (Comp.) sub-dimensions; OCI-r, Obsessive–Compulsive Inventory Revised; SCL-90 r OC, Symptom Checklist 90-Revised Obsessive-Compulsive subscale; SR, self-reporting; I, interview. Table reports comparisons between the baseline (t0) and follow-up (t1)*.

As regards the primary outcome, the Wilcoxon tests showed a significant decrease in the scores across t0 and at t1 on the Y-BOCS (SR) total self-report form (*p* < 0.003), and of both the related sub-dimensions, obsessions (*p* < 0.003) and compulsions (*p* < 0.003). All patients reported lower scores at t1. The descriptive statistics data analysis shows a decrease in scores at t1 compared to t0 for the specific measures for OCD: Y-BOCS (SR) total (t1: *M* = 16; *SD* = 8.7 vs. t0: *M* = 30; *SD* = 5.9); Y-BOCS (SR) Obs. (t1: *M* = 8.5; *SD* = 4 vs. t0: *M* = 15.2; *SD* = 2.8); Y-BOCS (SR) Comp. (t1: *M* = 7.5; *SD* = 4.9 vs. t0: *M* = 14.8; *SD* = 3.4); OCI-r (t1: *M* = 16.4; *SD* = 14.3 vs. t0: *M* = 21.5; *SD* = 15.5); SCL-90 r OC (t1: *M* = 1.2; *SD* = 1 vs. t0: *M* = 1.8; *SD* = 0.9).

In terms of the secondary outcomes, the Wilcoxon tests also showed a significant decrease in scores for OCD symptoms' severity assessed by means of the OCI-r (*p* < 0.026) and SCL-90 r OC subscale (*p* < 0.24). Thus, participants' scores of 9 (for OCI-r) and 8 (for SCL-90) demonstrated decreases at follow-up, indicating a significant treatment effect. Specifically, the Wilcoxon tests showed a decrease in scores for Y-BOCS (SR), OCI-s, and SCL-90 r OC for 11 participants (see Table 3).

Relating to the measures detected by psychotherapists, marginally significant improvements, and lower scores were found only in the Y-BOCS (I) Obs. (*p* < 0.045; 8 out of 11 patients had scores lower than at t1). There were no statistically significant differences in the total score and the sub-dimension of compulsions for Y-BOCS (I) (see Table 3).

However, although no significant differences emerge, qualitatively it is found that nine patients reported scores lower than t1 in the total Y-BOCS (I) score, while only 7 of 11 reported scores lower than t1 in the compulsions sub-dimension (see Table 2).

### Qualitative Data

From the qualitative data, collected from a questionnaire designed for this study, it is possible to observe good homogeneity of the sample. A total of 72.7% of participants were undergoing weekly treatment, the remainder being in treatment on a fortnightly basis (see Table 4). Only 1 of the 11 patients had suspended psychotherapy: 90.9% of the participants affirmed that their psychotherapy did not suffer suspensions.

**Table 4:** Descriptive data: responses to the qualitative questionnaire administered to OCD patients and their psychotherapists.

**Requested information**	**P answers %**	**T answers %**
CBT frequency of psychotherapy during pandemic and lockdown period	Yes: 90.9% No: 9.1%	Suspension: 9.1% Weekly: 72.7% Fortnightly: 18.2%
Evaluation of a worsening of symptoms	Not at all: 54.5% A little bit: 27.3% Moderate: 9.1% High: 9.1%	None: 63.6% Low: 18.2% Moderate: 18.2%
Evaluation of an improvement in symptoms	Yes: 90.9% No: 9.1%	None: 18.2% Low: 27.3% Moderate: 45.4% High: 9.1%
Perception of how much the psychotherapy in progress has represented a protective factor against a worsening or relapse	Yes: 100%	Not at all: 9.1% A little bit: 18.2% Moderately: 27.3% Quite a bit: 36.3% Extremely: 9.1

*P, patients; T, psychotherapist*.

However, as emerged from the therapists' questionnaire, some patients temporarily interrupted treatment, but it was just a summer interruption or was for work reasons.

More than half of patients (54.5%) did not observe a worsening of symptoms. Only in a single case did a therapist recognize the rise of a new obsession, probably falling into the checking subtype. This patient constantly checked if she was taking her birth-control pill correctly. In the other sample, therapists did not register the appearance of any new obsession or compulsion. In fact, 90.9% of the clinical sample noticed an improvement in their symptoms, and this was confirmed by 45.4% of the therapists, who claimed “moderate” progress in their patients.

All of the clinical sample affirmed that psychotherapy represented a protective factor against a worsening of their symptoms. According to these data, 45.4% of the therapists noticed a “quite a bit” or an “extreme” role of patients' psychotherapy during the pandemic.

## Discussion

This study aimed to investigate if the COVID-19 pandemic exacerbated the symptomatology of 11 OCD patients through an evaluation of the point of view of patients and psychotherapists. Indeed, this objective was pursued using psychodiagnostics evaluation carried out in two stages, with OCD patients and by recruiting their psychotherapists. This gave us the chance to consider evaluations regarding improvements/worsening of symptomatology and frequency of psychotherapy.

Although the sample was small, the results disconfirmed our hypothesis: patients did not have a worsening of symptomatology. We were still able to observe a significant decrease in the scores in the two specific OCD measures, Y-BOCS and OCI-r total scores, and in the SCL-90 OC subscale.

These statistical data were supported by the qualitative data wherein it was possible to observe a decrease in the follow-up scores compared to the baseline. Thus, despite this negative period, OCD patients' symptomatology was not adversely affected. The results also confirmed a decrease in symptomatology in self-report measures of the OCD re-test.

Thus, despite short periods of suspension of treatment, patients did not have worse symptomatology in the follow-up, as already highlighted by Kuckertz et al. (57).

For the qualitative data, as in many other studies (52, 53), we submitted questions to assess the subjective perception of the impact of the pandemic through the use of an *ad hoc* questionnaire. In line with others' results (56, 57, 97), we saw that most patients did not report a worsening of symptoms, and, in our study, the entire sample considered therapy a protective factor in the pandemic period.

Our observational study is one of the few studies (56) to assess symptom improvement in OCD patients during pandemic.

This result could be linked to the continued psychotherapy that the entire sample was receiving. The 11 patients were undergoing the same treatment procedure (67), an intervention procedure with CBT with ERP following international guidelines.

CBT with ERP treatment was used in two longitudinal studies (54, 57). In Kuckertz et al. (57), in response to COVID-19, part of the sample was more focused on the opportunity of engaging in new exposures and was interested in achieving new treatment goals. In contrast, in Storch et al. (54), clinicians observed a deterioration of symptoms in one-third of patients despite the continued therapy.

In our study, patients were followed for the same monitoring period and reported significant OCD symptoms at the baseline. Even though it is possible to observe lower scores in symptomatology in the follow-up from patients' results, in the psychotherapists' evaluations we detect only marginally significant improvements in obsession scores. Psychotherapists highlighted no statistically significant differences in the total score or in the compulsions sub-dimension of Y-BOCS. Despite this, even when not statistically significant, the global results of Y-BOCS were lower.

Thus, we observed a discrepancy between self-related and psychotherapist-related evaluations on the Y-BOCS, especially for the sub-dimension of compulsions. A similar result was highlighted by Hauschildt et al. (98). In that study, patients rated their own symptoms as less severe than their psychotherapists did, and the disagreement was stronger for obsessions and during the pre-assessment. These results could be linked to the inferior awareness that is typical in the early treatment stages, in which a patient does not really know the impact of the symptomatology.

These data have clinical relevance for OCD treatment. Despite the limited number of patients that did not allow us to conduct parametric statistical analyses, the results highlight how CBT procedure treatment (67) ensures a positive outcome. Although our results are not always statistically significant, the data that emerge are important from a clinical point of view and is in line with our clinical and research objectives, namely those of replicating the application of the OCD protocol and verifying the effectiveness of clinical practice.

In accordance with the naturalistic study of Mancini et al. (67) it is important to highlight in this study that patients undergoing CBT procedure treatment (67, 69) had some benefits despite the difficult period they were all enduring that could have contributed to worsening of the symptomatology, increase refusal of therapy or drop out.

It is important to point out that CBT associated with ERP is the gold standard of treatment for OCD (58). It has been proved that CBT with ERP is more functional than cognitive therapy and cognitive behavioral therapy, especially in the individual setting (60, 99, 100). As shown, patients undergoing CBT protocol treatment (67, 69) had some benefits despite the difficult period they were all enduring that could contribute to worsening of the symptomatology.

## Limitations

Despite our study bringing interesting results, our conclusions should be considered with caution, keeping in mind some important limitations. Firstly, our sample sizes for analyses were small; secondly, it was not possible to carry out parametric statistical analyses; and thirdly, no blinded heterodirect evaluation was conducted by other clinicians.

Moreover, we did not evaluate the presence of possible variables that could have moderated the symptomatology in positive terms. For example, being at home and had fewer opportunities to expose oneself to activating trigger.

## Conclusion

Our modest purpose was to monitor the symptomatic status of patients with OCD during the COVID-19 pandemic. Such a stressful period could have led to an exacerbation of obsessions and compulsions, and this made it necessary to obtain clinical data to use to build effective treatments.

Despite the small sample and the low statistical power of analysis, it is important to observe that our study used clinical data in two different monitoring periods and a specific intervention procedure. Our observational results highlight a global improvement in the symptomatology. These results were obtained using classic self-report measures for OCD and Y-BOCS, considered the gold standard for OCD assessment. In conclusion, it seems to us that a protocol that combines procedures with proven efficacy with interventions that emphasize the theme of acceptance of specific emotional and cognitive states, compared to treatment with ERP alone, produces greater normalization of symptoms and an improvement. We argue that interventions that target specific cognitive structures, ERP used as an intervention that facilitates acceptance of the risk of being guilty of irresponsibility, and increasing collaboration in the treatment have an effect on reducing OCD vulnerability. This highlights the importance of the specific procedure intervention to replicate the results obtained from empirical research, and is useful for clinicians to give clear effective indications for the treatment of OCD.

## Data Availability Statement

The raw data supporting the conclusions of this article will be made available by the authors, without undue reservation.

## Ethics Statement

Ethical approval was not provided for this study on human participants because all the subjects who were recruited signed the informed consent for any follow-up, collection, and publication of data for scientific purposes before undertaking the treatment, through documentation provided by the cognitive psychotherapy center in Rome. The study in question is an observational study. The request for publication of the data was submitted to the ethics committee. The patients/participants provided their written informed consent to participate in this study.

## Author Contributions

VZ, AG, and GF devised the procedure and carried out data collection. VZ wrote the manuscript with support from TC, MD'A, AM, and VP who analyzed the data. GF contributed to sample preparation. FM conceived the original idea and supervised the project. All authors contributed to the article and approved the submitted version.

## Conflict of Interest

The authors declare that the research was conducted in the absence of any commercial or financial relationships that could be construed as a potential conflict of interest.

## Publisher's Note

All claims expressed in this article are solely those of the authors and do not necessarily represent those of their affiliated organizations, or those of the publisher, the editors and the reviewers. Any product that may be evaluated in this article, or claim that may be made by its manufacturer, is not guaranteed or endorsed by the publisher.
